# Capturing learning from public involvement with people experiencing homelessness to help shape new physiotherapy research: Utilizing a reflective model with an under‐served, vulnerable population

**DOI:** 10.1111/hex.13397

**Published:** 2021-12-10

**Authors:** Jo Dawes, Duncan S. Barron, Laurence E. Lee

**Affiliations:** ^1^ Department of Epidemiology and Public Health, Collaborative Centre for Inclusion Health, Institute of Epidemiology and Health Care University College London London UK; ^2^ Department of Physiotherapy, Faculty of Health, Social Care and Education Kingston University and St George's University of London London UK; ^3^ Centre for Public Engagement, Faculty of Health, Social Care and Education Kingston University and St George's University of London London UK; ^4^ Department of Neuromuscular Diseases, UCL Queen Square Institute of Neurology University College London London UK

**Keywords:** health service research, patient and public involvement, people experiencing homelessness, physiotherapy, reflection, under‐served, vulnerable

## Abstract

**Introduction:**

People experiencing homelessness (PEH) have poorer health than housed people but face barriers accessing care and being involved in research. As an often‐ignored group, their contribution to help shape research that is for and about them is essential, as it can strengthen the research proposal, in turn facilitating research and outcomes that are relevant to this vulnerable group.

**Methods:**

Six people with experience of homelessness attended a PPI consultation aided by Pathway, a UK homeless peer advocacy charity, which coordinates an ‘Experts by Experience’ group. We present reflections on conducting PPI with PEH that informed the development of a physiotherapy research proposal. Kolb's Experiential Learning Cycle guided reflections across four stages: (1) describing the PPI experience; (2) reviewing and reflecting on the PPI experience; (3) learning from the PPI experience; and (4) planning and trying out learning.

**Results:**

Reflections highlighted the importance of: working closely with an advocacy organisation and leader to reach under‐served people; the diversity of experiences; using familiar venues, contingency and budget planning; flexibility and ‘allowing time; talking less; listening more’; planning for early and ongoing PPI, and the potential of mobile ‘one‐off’ PPI outreach models to reach vulnerable groups.

**Conclusion:**

Kolb's Experiential Learning Cycle aided team reflection on feedback from PEH, which helped refine and strengthen a physiotherapy research proposal. The project was unfunded. However, a reflective model helped maximize learning and impact including for future PPI and research. The novel application of Kolb's Experiential Learning Cycle provided structure, facilitated reflection and enhanced individual and collective learning and may benefit capturing learning from PPI with other vulnerable populations.

**Patient or Public Contribution:**

We highlight how a PPI consultation with people with experience of homelessness helped shape a funding proposal. Additionally, the reflections of the experts by experience team leader are included.

## 1 INTRODUCTION

### 1.1 Aims and objective

This reflective case study shares individual and collective learning from patient and public involvement (PPI) activities focussed on the development of a mixed‐methods research funding proposal investigating accessibility, acceptability, and the need for physiotherapy services for people experiencing homelessness (PEH). We reflect on how involving vulnerable and under‐served PEH in a new physiotherapy study helped inform both the funding proposal and future research planned by team members. We aim to impart learning to help inform researchers undertaking PPI with other underserved and vulnerable groups.

### 1.2 PEH and their health

A person experiencing homelessness can be defined as anyone who has no home^1^ and includes people identified in official United Kingdom (UK) statistics as ‘legally homeless’ and the ‘hidden homeless’ (those out of sight in squats, hostels, Bed and Breakfasts or staying with friends). In England alone, the most extreme forms of homelessness^2^ have increased year on year since 2010^3^ with the latest estimates standing at 280,000 people.^4^ During the 2020 pandemic the UK government announced the ‘Everyone In’ scheme, housing around 29,000 rough sleepers in emergency accommodation^5^ and guaranteed residential tenants protection from eviction during this period.^6^ Both may have positively impacted homelessness statistics. However, economic challenges resulting from the pandemic may increase homelessness when such interventions no longer apply.

PEH have poorer health than housed people, often experiencing a ‘tri‐morbidity’ of mental and physical ill‐health, substance misuse^7^ and early mortality.^8^ The combination of ill‐health, coupled with insecure living arrangements, can make PEH some of the most vulnerable in our society.

The incidences of musculoskeletal, cardiorespiratory problems and brain injury^9^, ^10^, ^11^ are greater amongst PEH than in the general population. Care of these conditions is the core area of physiotherapy practice, with rapid access recognized as vital in preventing more severe or long‐term health problems.^12^ Despite a high prevalence of these conditions amongst PEH, research shows they have difficulty accessing National Health Service (NHS) physiotherapy.^13^ General practitioner (GP) referral is the main access route to NHS physiotherapy care. However, around 33% of people sleeping rough are not registered with a GP, compared with only 2% of the English general population.^14^ Barriers to registering and attending primary care include frequently moving areas, a chaotic lifestyle and lack of transport.^14^ These challenges may explain why PEH are regarded as being under‐served by current healthcare provisions.

### 1.3 The UK Research Governance Framework: Involving vulnerable people in research

Researchers aiming to improve the health of vulnerable and under‐served groups must ensure their voices are heard throughout the research process. Current UK guidance states that health and social care research must provide an environment where patients, service users and the public ‘…are given, and take, the opportunity to participate in health and social care research and to get involved in its design, management, conduct and dissemination, and are confident about doing so’.^15^

### 1.4 Patient and public involvement in research with vulnerable, under‐served populations

Patient and public involvement (PPI) in research is defined as ‘… research being carried out “with” or “by” members of the public rather than “to”, “about” or “for” them’.^16^ The founding principle being, people who are affected by research have a right to have a say in how it is designed, undertaken and disseminated.^17^

An earlier version of UK research guidance explicitly referenced the importance of involving PEH in research.^18^ Vulnerable members of the public also include children and young people, older people, people from low socioeconomic backgrounds, prisoners, those living with chronic illnesses, people from black and minority ethnic backgrounds and asylum seekers.^19^ Such groups tend to have greater health care needs than the wider population^20^ and face multiple barriers in engaging with healthcare services, further compounding health problems.^14^ They are also frequently excluded from involvement to help shape and deliver research.^21^

‘Under‐served groups’ is a term adopted recently by the NIHR's INCLUDE guidance^22^ in preference to others (e.g. ‘hard to reach’) that erroneously imply the lack of inclusion is due to the fault of the members of these groups. There is no single definition of under‐served groups, but characteristics common to them include: lower inclusion in research; high healthcare burden unmatched by the volume of research designed for the group and differences in how they respond to or engage with healthcare interventions compared to other groups, with research often neglecting to address these factors.^22^ We use the term ‘under‐served’ to refer to PEH in terms of their access to both health services and research opportunities, as it is a useful reminder that healthcare and research communities need to engage better to provide appropriate services and opportunities for these groups. Additionally, their views on improving the accessibility of services are likely to go unheard.

Researchers must explore opportunities to undertake PPI when planning and prioritizing research about health services for vulnerable, under‐served groups, and reflect on those opportunities to help support wider learning about how involvement works, for whom and when.^23^

### 1.5 Reflexivity in PPI work

Developed against a backdrop of debates around measuring PPI impact,^24^ there is recognition that reflecting on patient and public activities can help capture researchers' ‘rich and valuable’ subjective accounts of the value and impacts of PPI.^23^ It is argued that reflexivity forms one of the fundamental principles underlying the evaluation of PPI.^25^ Staley and Barron^23^ suggest reporting of PPI should enable researchers to ‘tell the story’ of what happened, ‘…where the researchers started, what they learnt from their conversations with the public…’ and ‘…what changed as a result…’.^23^ Reflecting on PPI activities may help researchers to learn from, and impart their PPI story. The NIHR UK Public Involvement Standards encourages researchers to reflect on the PPI they undertake though no guidance is given regarding how.^16^

Previous reports of reflections of PPI have adopted ‘critical reflection’ techniques using written and annotated diaries employed in different degrees by various members of the research team, including: researchers^26^ only; researchers and public contributors reflecting separately^27^ and, collective reflections of the entire team.^28^ Techniques drawing on ‘models’ of reflection have also been applied to learning from PPI activities. For example, a reflective model developed by Marks‐Maran and Rose,^29^ which comprises four components (the incident; reflective observation; related theory and future action) was used to reflect on the PPI experiences of a research team developing a stroke study.^30^ However, we chose The Experiential Learning Cycle^31^ to reflect on our PPI activities due to its established utility within physiotherapy and familiarity within the team.

### 1.6 Context

This project was led by J. D., a physiotherapist with extensive clinical experience in homelessness healthcare. It was designed to inform a proposal for research exploring physiotherapy services for PEH. Research priorities in healthcare for PEH have recently been identified using a stakeholder event. However, it did not involve people with lived experience of homelessness.^32^ The challenges of involving PEH in PPI and research have recently been discussed, highlighting the complications of paying appropriate fees to people in receipt of welfare benefits, or the difficulty some people might have in committing to a project of long duration (perhaps over many years).^33^ These insights further show the importance of our work to help to fill a knowledge gap.

Our paper highlights the team's experiences and reflections of involving PEH in the design of a new physiotherapy study using a reflective model routinely used by physiotherapists in training and practice.

## 2 METHODS

### 2.1 Reflections of undertaking PPI using the Experiential Learning Cycle

Within physiotherapy, reflective practice has commonly been taught utilizing Kolb's Experiential Learning Cycle^31^ to review experiences and identify action to bring about change in practice.^34^ Its value and applicability may not be limited to physiotherapy and may offer a useful framework for others to learn from their PPI experiences regardless of backgrounds. Kolb's is a cyclical process involving four stages (we have modified the wording slightly to incorporate ‘PPI’):

1. Concrete experience (describing the PPI experience).
2. Reflective observation (reviewing and reflecting on the PPI experience).
3. Abstract conceptualisation (learning from the PPI experience).
4. Active experimentation (planning and trying out what has been learned).

### 2.2 Stage 1: Concrete experience (describing the PPI experience)

We describe two experiences pertinent to this reflective case study. First, undertaking a PPI consultation with PEH and secondly, undertaking the process of reflection.

#### 2.2.1 Undertaking a PPI consultation with PEH

A research idea and question were formulated building on previous research looking at access to mainstream NHS physiotherapy for PEH.^13^ It was important to discuss the new ideas with people who had experienced homelessness, to establish whether the research question was relevant to them and to gain insights regarding the proposed recruitment, data collection, analysis and dissemination methods.

The research team engaged with Pathway, a leading UK homeless peer advocacy charity to help reach people with lived experience of homelessness.^35^ Pathway runs Experts by Experience (EbE), a group led by and for people who have experienced homelessness. EbE aims to influence healthcare providers, commissioners and researchers to ensure services and research are designed to best meet the needs of people experieincing homelessness.^36^ The EbE team leader (who also had lived experience of homelessness) helped recruit EbE members to attend a PPI consultation to help inform the funding proposal.

The PPI consultation was held in a room used by Pathway, led by J. D. (Principal Investigator and senior lecturer in physiotherapy) and supported by coinvestigators D. B. (an expert in involvement) and L. L. (newly qualified physiotherapist embarking on a research career). The EbE Team Leader (S. B.) recruited six people with lived experience of homelessness to attend the session who were diverse in age, gender identity and nationality. Some also had additional experiences of: addiction and mental and physical health difficulties; living through the care system; insecure accommodation; being housed and some were now employed. Attendees were briefed about the safe disclosure of information and recompense for travel costs.

The session ran from 10.00 until 14.00, commencing with tea and coffee, introductions and an overview of the session. The group agreed to hand‐written notes (taken by D. B. and L. L.) and an audio recording. The session comprised three parts:

1. *Exploration of the experience of homelessness and individuals' perceptions about the relevance of physiotherapy care to them*: J. D. asked attendees about their own experiences of physical health problems, how they had sought help in addressing them and any experiences of physiotherapy. Vignettes and case studies from J. D.'s clinical experience of working with PEH were used to initiate discussions about health problems that physiotherapy can address. This provided a platform for people to discuss personal experiences or those of others they had observed interacting with NHS physiotherapy while they were homeless.
2. *Discussion about the proposed research*: J. D.'s ideas about methods were presented to the group who were invited to comment, critique and suggest alternative ways to answer the research questions. Focus was placed on the recruitment of PEH as research participants, how they could be interviewed and challenges that may be encountered with data collection.
3. *Discussion about establishing an advisory group and ensuring meaningful PPI throughout the project*: This session benefited from the establishment of trust and openness that working together in the two earlier sessions had facilitated. There was an open discussion covering the topics of frequency of advisory groups and PPI session, how they can effectively operate and how to optimize the involvement of PEH.

The session included a one hour lunch break during which a meal was provided. Costs of refreshments, travel and ‘thank you’ vouchers were met from the NIHR Research Design Service London Enabling Involvement Fund.^37^

#### 2.2.2 Undertaking the process of reflection

The process of reflection occurred over two time periods. The early reflection took place within a week of the EbE consultation. This focussed on how the PPI could inform the funding application. A debrief meeting was held with the coinvestigators during which they reviewed and reflected on the consultation notes and audio recording. J. D. then adapted the research proposal before submission (described in J. D.'s reflections).

Further reflection occurred after the (unsuccessful) NIHR funding outcome was known. The consultation notes and recording were revisited by the team who were keen that, despite the unsuccessful funding outcome, learning from the PPI session should not be lost. The latter focus was on how the PPI consultation helped inform team members' subsequent PPI thinking and research activities. We recorded our collective learning, recognizing this may be transferable to PPI with other under‐served or vulnerable groups. We individually followed the four phases of the Experiential Learning Cycle^31^ to aid reflections. J. D. collated and summarized key topics, which were circulated within the team for further debate and clarification, until finally a consensus was reached on key collective learning outcomes.

## 3 RESULTS

### 3.1 Stage 2 reflective observation (reflecting on the PPI experience) and Stage 3 abstract conceptualisation (learning from the PPI experience)

Team reflections are first presented individually, followed by key points from our collective learning, which were identified during group discussions.

#### 3.1.1 Reflections and learning from physiotherapy senior lecturer and principal investigator (J. D.)

##### Reflections informing the funding proposal

Undertaking the PPI session helped improve the research proposal. The EbE provided many examples of times they had struggled to access physiotherapy services, reinforcing the importance of the proposed research topic. Moreover, J. D. was able to make important changes to the proposal in response to feedback. For example, the role of site ‘advocates’, (i.e., a person or people with experience of homelessness who could assist in recruiting other people living in similar circumstances) was added to the proposal. The EbE also highlighted the importance of flexibility if PEH are to be included in advisory groups—pointing out that their circumstances can be unpredictable and change quickly.

##### Reflections informing future PPI with under‐served/vulnerable groups

J. D.'s wider reflections of undertaking PPI with PEH focused on her organisation of the PPI session and her need to reach a range of PEH within a limited budget and time frame. She found collaborating with EbE invaluable and appreciated the support of S.B., the EbE Team Leader.

J. D. was new to PPI and approached the planning of the session in a very structured way. She adopted a consultative approach to the session, which assured her that important topics would be discussed in the time available. Conversely, during the process of reflection, she recognized this researcher‐led approach potentially limited what she found out from this experience‐rich group of people. It highlighted the need for a more collaborative approach, where handing over greater control and involvement to the PEH and seeking their priorities earlier within the PPI process may have allowed for greater input from them; something she was keen to put into practice in future PPI work.

J. D. felt grateful to have a route to reach PEH and was in no doubt that working with an advocacy group like EbE provided access to people that she would not otherwise have been able to contact. J. D. recognized that relinquishing control of aspects of organizing the PPI session and being flexible required trust that all would go to plan. She had confidence in the leader of EbE to arrange the meeting as agreed but also recognized that some details were informal and could change at short notice, including nonattendance. With no alternative arrangements agreed, this was recognized as a threat to the success of the session. The reflective process helped J. D. recognize that in the future she would have clearer discussions with any third‐party organisation about troubleshooting unanticipated events and have clearer agreements about how to manage them.

#### 3.1.2 Reflections and learning from the newly qualified physiotherapist (L. L.)

L. L. came to the PPI session as a newly qualified physiotherapist and early career researcher. With no previous involvement in PPI, he saw this as an opportunity for learning and reflection. During the PPI consultation, he took on the responsibility of note‐taker, documenting the discussions in detail. He purposefully adopted the role of an active listener, which involved maintaining moderate to high nonverbal involvement, while also, when appropriate, paraphrasing the speaker's message and asking clarifying questions.^38^ In taking this approach, he helped enable the voices of individuals from EbE to be respected and heard.

L. L. identified and interpreted his reflections around two key areas: physiotherapy provision for PEH and the PPI process itself. The EbE consultation session highlighted how prevalent conditions that physiotherapists commonly treat were for these people. He reflected on his recent undergraduate training and how the holistic skills of physiotherapists could benefit this population. He contrasted this with the recognition that unconscious biases of the profession could well contribute to a lack of access for vulnerable groups such as PEH. His thoughts on PPI activities were also moulded by this experience. He gained a greater understanding of the importance of PPI and recognized that the public and patients should be more involved in research.

#### 3.1.3 Reflections and learning from an expert in public involvement (D. B.)

As a member of the PPI team with substantial experience of advising on and undertaking public involvement, D. B.'s reflections focused more on considering his experience of PPI with what the team could take from the EbE session. He noted that PEH seemed to lack understanding of the health benefits afforded them by physiotherapy. Therefore, spending some of the early parts of the consultation session to explore physiotherapy benefits for PEH was very important. He noted the value the EbE placed on advocacy roles, in particular, ‘peer advocacy’. For example, the EbE recommended that PEH are invited to assist with participant recruitment or data collection. D. B. reflected that ‘peer advocacy’ could also be adopted when recruiting public contributors to take part in PPI consultations; something that might help reach under‐served, vulnerable groups. He also recognized the importance of the ‘guardian role’ the EbE Team Leader fulfilled in bringing PEH together and the importance of the session being hosted in a space familiar to the EbE. He believed both of these promoted trust and good working relationships between the EbE and the research team, resulting in the success of the consultation session.

D. B. valued the insight the consultation provided on how to reach vulnerable, under‐served people and how this new knowledge could be applied when conducting PPI for future funding applications with similar groups. For example, the EbE placed great importance on healthcare being available and accessible for PEH, highlighting that services for this population often don't have positive impacts unless they are genuinely ‘outreach’ in their approach (e.g., the mobile ‘TB van’). D. B. was struck by how the EbE were interested in providing continued PPI input as the project developed and many wished to join an advisory group for future research activities, despite the many challenges they faced in their lives.

In supporting J. D. with her funding application, D. B. observed the positive impact the EbE PPI consultation had on informing and influencing change in her research and was encouraged when feedback about the PPI undertaken for the proposal was regarded as ‘strong’ (the highest category) by all the NIHR reviewers.

#### 3.1.4 Reflections and learning from an  EbE (S. B.)

S. B. took responsibility for: inviting PEH to the PPI consultation session; managing the administration of recompensing travel expenses, ‘thank you vouchers’ and lunch costs from the PPI funding and booking a suitable venue for the session. He supported attendees to get to the venue and checked they had received travel claim forms and thank you vouchers. As a person with lived experience of homelessness, S. B. participated in the PPI discussions, but also considered himself a ‘protector’ and advocate of his EbE colleagues, ensuring all involved were clearly briefed about safe disclosure of their personal information and ‘stories’. He knew the attendees well and was satisfied they presented a range of diverse backgrounds and experiences (e.g., in terms of age, gender identity, nationality, experiences of addiction, mental and physical health difficulties and their varied experiences of homelessness, which included rough sleeping, sleeping in cars, hostel, B&B, temporary flat and permanent accommodation). On reflection, he described his beliefs about the importance of researchers attending PPI consultations with open questions and stressed the importance of ‘listening to allow people's stories to come to life’; something he acknowledged was done well in the PPI session described.

S. B. stated the importance of allowing time for PPI activities and how he observed people's confidence increased as they spoke. He felt the more time conversations had to evolve; the more people opened up. S. B. believed that how researchers conducted themselves was important to the success of the sessions and that they can leave PPI sessions with vulnerable groups having more questions than answers. A phrase he used in his reflections was a ‘slow boil’, suggesting that slowing down the discussion facilitated trust and confidence, thus enabling the group to work well together. He described the work involved in supporting vulnerable people with complex experiences to attend, stating ‘the bus doesn't just turn up’. He suggested that those given the opportunity to tell their story benefitted when they felt heard, but with that comes vulnerability. He recommended that such vulnerability should be acknowledged and valued by the research team.

## 4 SUMMARY OF COLLECTIVE PPI LEARNING FROM OUR CONSULTATION REFLECTIONS WITH EBE

Sharing, collating and synthesizing personal reflections on the PPI consultation within a team provided an enhanced depth of understanding. For example, each team member reflected on different aspects of what they took from the PPI session, but when this was shared, the group understanding, learning and development of ideas expanded for all members. L. L., for example, recognized the link between S. B. describing slowing conversations down ‘talking less and listening more’ and his experience of choosing to be being an ‘active listener’. This resonated with J. D., to the extent that she has applied it in her future research plans. A summary of our collective learning from our reflections is outlined in Table 1.

**Table 1:** Collective reflective learning from PPI with PEH

Theme	Identified by	Collective learning	How can the learning be applied?
Working with advocacy organisations and leaders	D. B., S. B., J. D.	Leaders of advocacy organisations may hold a protective, guardianship and advocate role with their members. An advocate can be important to help establish relationships and rapport, enabling people to experience safe boundaries a safe space to disclose personal experiences. An advocate or ‘protector’ can help ensure ethically sensitive PPI being conducted. Employing peer advocates may help recruit to PPI and aid the diversity of those invited. Advocated may also help recruit research participants	Researchers should consider the vulnerability of under‐served groups and the value of their disclosure of information. Third‐party organisations and community leaders trusted by target populations can not only help to maximize reach but also provide support, protection and advocacy for vulnerable people thereby fostering a safe, ethical PPI space. Consideration should be given to employing advocates to help recruit under‐served populations to both PPI consultations and research studies (as participants). Researchers should budget to pay peer advocates to support PPI and research activities
Diversity	J. D., S. B.	Diversity of backgrounds and experiences (in addition to the experience of homelessness) among individuals taking part in the PPI group enhanced the depth and variety of discussion	Clinicians or researchers embarking on PPI with under‐served groups should consider how they can achieve diversity particularly if they aim to engage under‐served populations possibly with help from community leaders/advocates
Venue, contingency planning and budget	D. B., J. D.	Utilizing a meeting venue familiar to the PPI contributors may help promote trust and establish relationships among attendees that aid working together and learning. It is important to discuss and formalize arrangements, including contingency planning and ‘trouble‐shooting’ in case things do not run as expected. Reimbursing travel and expenses is also important for under‐served groups.	Anyone keen to reach vulnerable or under‐served groups should choose a location or venue that is accessible, familiar and easy to get to for the target population. Teams may need to factor contingency planning into PPI plans. Plan for the unexpected Budget for PPI with under‐served, vulnerable groups
Flexibility and time: ‘Allow time; talk less; listen more’	J. D., L. L., S. B.	Research teams should avoid overly rigid or inflexible consultations. They should attend with questions, but also with an openness and flexibility to respond to what is heard from the experts by experience. In other words, researchers should ‘allow time, talk less, listen more’ to enable peoples' ‘stories to come to life’. Active listening can help clarify any points raised and foster respect. Vulnerable people may need time (e.g., the ‘slow boil’) to openly share stories	Early career researchers or people with limited experience of carrying out PPI may feel they need to heavily structure PPI activities to feel confident that all information gathering will be covered. However, flexibility, allowing time and a slow pace and listening are extremely important to ensure stories are heard
Early and ongoing PPI	L. L., D. B.	Researchers should value the importance of PPI input from the target population throughout the research process, not just at the beginning. Early involvement may help establish trust	Evidence of early predesign PPI is often a stipulation of a funding bid, but researchers should be mindful that it is also vital to help inform throughout the duration of a project, not just the early planning stage
Mobile ‘one‐off' outreach PPI	D. B.	Mobile ‘one‐off’ PPI outreach approaches modelled on services targeting vulnerable groups’ needs (e.g., TB vans for PEH) could benefit both PPI and research recruitment	Clinicians and researchers should learn from existing, ‘trusted’ outreach services that are already successful in reaching under‐served people. Truly mobile and outreach PPI consultations (perhaps using vans or ‘pop up’ venues) could be trialled with under‐served groups as they may help break down barriers to involvement, make attendance more convenient and broaden the reach beyond the ‘normal suspects'

*Note*: J. D.—Experienced physiotherapist, early career researcher. L. L.—Newly qualified physiotherapist, embarking on doctoral‐level research. D. B.—Experienced researcher and expert in PPI. S. B.—Lead of organisation for reaching experts by experience.

### 4.1 Stage 4: Active experimentation (planning and trying out what has been learned)

Stages 1–3 of the Experiential Learning Cycle^31^ enabled members of the team to plan and try out learning from their reflections, through informing future PPI work for new research projects.

The process of reflection resulted in J. D. and L. L. both applying their learning to their future research. L. L. recognized his involvement in the PPI session had helped shape him as a researcher and sparked his interest in more participatory design methods. For example, his current doctoral research now includes an evaluation of a coproduced self‐management programme and monitoring of his research processes, as well as coproduced dissemination through video blogging and creative public engagement approaches. The inclusion of which was influenced by an appreciation for the significance of collaboration, and the possibilities for overcoming established power structures that stemmed from his reflections. J. D. has since adopted a more reflective practice as part of several new research projects and found continued benefits of reflecting (including with others) in helping her and colleagues achieve an improved understanding of all aspects of the research process. J. D. also felt reflection highlighted a greater appreciation of the importance of involving service users early in research design, motivating her to consider more participatory research methods, such as experience‐based codesign,^39^ in her future research.

## 5 DISCUSSION

The PPI session and subsequent reflections reported in this paper demonstrate the value of reaching vulnerable, underserved groups to inform new research, which affects them and the potential value of the reflection and learning process for teams undertaking PPI with similar groups. Vulnerable groups, such as PEH, are known to struggle to access healthcare services like physiotherapy.^13^ It is also vital they are not overlooked by teams planning research that affects them; something we hope our reflections help address.

A recurring observation from the collective reflections was the value of working with a third‐party advocacy organisation and community leader to reach a diverse group of PEH. The research team did not have direct contacts with PEH, nor resources to build links on the ground, so it offered pragmatic advantages for engaging potentially people from a socially disadvantaged group, an important issue highlighted in a related systematic review.^40^ As others have noted, involving public contributors early and undertaking reflections together may help foster a collaborative approach whereby relationships and trust are built and maintained between researchers and public contributors,^41^, ^42^ helping address power differentials.^43^ Collaborating with a community representative external to the research team has also been recommended elsewhere^42^, ^43^ as this provides a useful ‘buffer’ should public contributors have concerns that may be difficult to raise directly with the research team. The ‘guardian role’ adopted by the EbE Team Leader in our project may have helped foster ‘ethically conscious’^44^ PPI being undertaken, by helping our vulnerable group of people with experience of homelessness feel safe and able to speak openly. Involving members of the community of interest as advocates (or who adopt ‘protective roles’) may help the conduct of more ethical PPI from the outset and help reach a broader more diverse population; something the EbE leader felt had been achieved in this project.

Holding the PPI consultation at a venue hosted by pathway may have afforded a well‐known and safe environment, enabling attendees to discuss their views and experiences openly. As other commentators have stressed, budgeting to enable public contributors to attend a venue and to cover out‐of‐pocket expenses is crucial,^42^, ^43^, ^45^, ^46^ and was offered to our attendees.

The EbE Team Leader's reflections emphasized additional important lessons for the team when working with PEH. He stressed the need for research teams to be flexible, listen and allow time for stories to emerge (i.e., the ‘slow boil’). He also emphasized the need for researchers to enable under‐served people's stories to ‘come to life’ by: ‘allowing time, talking less, listening more’. Allowing insufficient time has been commented on by others as contributing to suboptimal, unethical PPI and is potentially burdensome to public contributors.^44^, ^45^, ^46^ The value of embedding PPI as early as possible into new and ongoing research proposals was also a lesson highlighted in our reflections and which chimes with previous projects.^42^, ^45^ The apparent willingness of those involved in our consultation to be involved in future PPI activities despite their changeable life circumstances was encouraging.

The consultation with PEH highlighted the importance to them of health services being available and accessible to them. They stressed that to be effective, services need to be genuinely ‘outreach’ in their approach. This could be adapted and applied to PPI with vulnerable groups. One‐off, ‘mobile’ PPI sessions have previously been conducted at pre‐existing venues^47^ and could be further expanded for vulnerable, under‐served groups. Truly mobile (i.e., in a van or bus) outreach PPI, may help expand the service user volunteer pool beyond the ‘usual suspects’ and be particularly convenient when undertaking PPI with PEH and other vulnerable groups. A mobile outreach van would not initially be a venue well‐known, but with time and collaboration with community leaders/advocates, mobile outreach PPI may become as trusted as any other ‘venue’ frequented by under‐served groups and is therefore worth considering in future projects.

Feedback from PEH and lessons highlighted in collaboration with the EbE Team Leader underscores that PPI is a two‐way learning process.^23^ Researchers can learn from the lived experiences of members of the public about how PPI consultations and research can be designed and undertaken. ‘Working together’ in a way that ‘…values all contributions, and that builds and sustains mutually respectful and productive relationships’ was a strength of this study and forms one of the six UK Public Involvement Standards^16^; a framework to help researchers and members of the public identify what good public involvement in research looks like. The Standards explicitly encourage ‘reflection and learning, including where lessons have been learned…’. However, no guidance or advice is offered on how researchers or public contributors undertake their reflections.

Some authors have reflected on PPI utilizing diary techniques,^26^, ^27^, ^28^ or personal written reflections^41^ while others have used interviews and workshops.^42^ One of the few studies to have utilized an existing model to reflect on PPI activities adopted Marks‐Maran and Rose's ‘Reflection Cycle’, which contains four components: (i) the incident (ii) reflective observation (iii) related theory and (iv) future action,^30^ and closely aligns with Kolb's reflective cycle. Although we did not aim to compare these models in practice, future research could do so and include others. For convenience, we simply employed an existing reflective model used commonly in physiotherapy practice and familiar to the research team. Kolb's model may also be useful for researchers from other clinical backgrounds to help reflect on and evaluate PPI activities. Using reflective models such as Kolb alongside the UK Standards could help teams evaluate how well they achieve any of the six standards so that learning can be maximized.^34^, ^47^ Although we did not evaluate how well we achieved any of the standards, there are indications that trust and respect and therefore meaningful PPI resulted.

Previous studies have undertaken different approaches to reflecting on PPI primarily at the end of their studies.^30^, ^41^, ^43^ We undertook ours in‐depth early on at the design stage. We recommend the reflective practice and learning taking place at the earliest opportunity (as well as throughout the study) to maximize impact on shaping the proposal and delivery of research. Reporting of PPI rated highly by funding reviewers, but which may ultimately be unfunded should still be routinely reported so that learning can be maximized and not lost.

A strength of this paper is the novel application of Kolb's Experiential Learning cycle^31^ to aid reflection, and to our knowledge, this is the first time Kolb has been used to reflect on and evaluate PPI. Using the four stages of Kolb^31^ to facilitate reflections on PPI was a pragmatic and relevant approach for a team that included physiotherapy researchers since the model is widely used in physiotherapy education and clinical practice.^49^ By including all members of our research team and an EbE in the reflection phase, and supplementing this with team discussions, we aimed to mitigate the power imbalances that often exist in research teams via dialogue.

A limitation of the Experiential Learning Cycle model is that it may fail to account for how ‘reflection in action’ can be utilized as a learning tool.^50^ Therefore, we advocate supplementing the model with team reflections (including members of the public) that are dialogical rather than discursive, to consider collective thoughts on the shared PPI experience. This paper demonstrates the benefit of including an EbE in the reflection process and the value it brought to the teams' understanding of the experience.

## 6 CONCLUSION

The individual and collective PPI reflections and learning reported in this paper were inspired by an early‐stage PPI session with a vulnerable group, with the aim of informing a research funding application. This paper demonstrates the value of undertaking PPI with vulnerable, under‐served groups such as PEH, and how individual and team reflections using an existing model have the potential for enhancing the learning from PPI undertaken with other vulnerable groups. The planned physiotherapy research project was ultimately unfunded. However, by using a theoretical model to reflect on the early design stage PPI process we have gone some way to optimize learning both individually and collectively from the PPI experience. Reflecting on and getting the most out of PPI activities is an ethical imperative, even when projects are not funded.^43^ We concur that ‘…a reflection cycle can be usefully employed as a tool to structure ongoing dialogue about the motivations, contributions and experiences of researchers and members of the public when they work together to develop and conduct health research studies’.^30^

## CONFLICT OF INTEREST

The authors have declared no conflict of interest.

## ETHICS STATEMENT

This data within this paper was not generated from primary research, rather the authors' reflective account of a PPI experience. Therefore, no ethical approval was needed.

## Data Availability

The data that support the findings of this study are available from the corresponding author upon reasonable request.
